# Are we ready to design oral PROTACs®?

**DOI:** 10.5599/admet.1037

**Published:** 2021-08-31

**Authors:** Diego Garcia Jimenez, Matteo Rossi Sebastiano, Giulia Caron, Giuseppe Ermondi

**Affiliations:** University of Torino, Molecular Biotechnology and Health Sciences Dept., CASSMedChem, via Quarello 15, 10135 Torino, Italy

**Keywords:** Degrader, bRo5, Bioavailability, Permeability, Chemical space, PROTAC-DB, Chameleonicity

## Abstract

PROTACs® are expected to strongly impact the future of drug discovery. Therefore, in this work we firstly performed a statistical study to highlight the distribution of E3 ligases and POIs collected in PROTAC-DB, the main online database focused on degraders. Moreover, since the emerging technology of protein degradation deals with large and complex chemical structures, the second part of the paper focuses on how to set up a property-based design strategy to obtain oral degraders. For this purpose, we calculated a pool of seven previously ad hoc selected 2D descriptors for the 2258 publicly available degraders in PROTAC-DB (average values: MW= 972.9 Da, nC= 49.5, NAR= 4.5, PHI= 17.3, nHDon= 4.5, nHAcc= 17.7 and TPSA= 240 Å^2^) and compared them to a dataset of 50 bRo5 orally approved drugs. Then, a chemical space based on nC, PHI and TPSA was built and subregions with optimal permeability and bioavailability were identified. Bioavailable degraders (ARV-110 and ARV-471) tend to be closer to the Ro5 region, using mainly semi-rigid linkers. Permeable degraders, on the other hand, are placed in an average central region of the chemical space but chameleonicity could allow them to be located closer to the two Arvinas compounds.

## Introduction

Traditionally, the search for novel treatments has been undoubtedly driven by small molecule discovery. However, since the release of Muromonab in 1986, biologics have gained importance. Even though the market impact for this class of molecules was predicted to rise faster, 71 % of 2020s approved drugs (38) correspond to small molecules which still assume, 35 years later, the burden of drug discovery research (www.fda.gov). Moreover, the guidelines for small molecule development have evolved from the traditional agonism-antagonism concept to innovative mechanisms of action. Thus, small molecule research has enlarged its scope to nucleic acid-based modalities, protein-protein interaction (PPI) modulators, peptide/peptidomimetics and PROTACs® or degraders [1].

**Figure 1.**
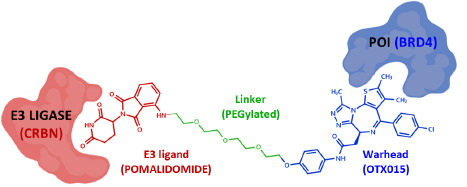
Structure of degraders and their building blocks (ARV-825 is shown as an example).

In particular, PROTACs® have been revealed to be a promising therapeutic area, which has expanded from 13 scientific articles in 2016 to 265 in 2020 (www.pubmed.com). Structurally, PROTACs® are heterobifunctional degraders made of a warhead binding a protein of interest (POI), a ligand recruiting an E3 ligase and a linker coupling both regions (Fig.1) [2]. Biochemically speaking, their mechanism of action involves the formation of a ternary complex (E3 ligase:PROTAC®:POI) in which the E3 ligase triggers the ubiquitination and subsequent degradation of the POI by the ubiquitin-proteasome system (UPS) [2].

Unlike traditional small molecules, based on occupancy pharmacological effects, degraders make use of this innovative event-driven mechanism of action, presenting several advantages over conventional strategies. This is considered a catalytic and sub-stoichiometric event, which allows the targeting of several units of the POI with just one PROTAC® molecule [3]. In addition, due to its event-driven pharmacology, the degradative activity of the UPS does not require the highest affinity of the warhead for the POI [4]. Therefore, unlike traditional inhibitors requiring higher affinities, degraders are able to act on proteins thought to be ’undruggable‘, which expands the therapeutic scope from protein-dependent diseases to viral infections, cancer and immune and neurodegenerative disorders [5]. Nonetheless, PROTAC technology is still in its infancy and is not yet completely understood [6].

Degraders, first introduced by Crews and Deshaies in 2001 [7], have now a few candidates in clinical trials. In fact, ARVINAS announced in the first half of 2020 the first two oral degraders to reach Phase 2 clinical trials (https://www.arvinas.com). Moreover, the number of PROTACs® in the clinic should rise to 15 at the end of 2021, which reveals a higher interest in this field and a better understanding of their functioning [8]. However, despite their promising pharmacodynamic potential, the cautious introduction of PROTACs® into the market may also be explained by their poor bioavailability. Moreover, this limitation is often related to drug metabolism and pharmacokinetics (DMPK) challenges, such as water solubility and permeability issues, derived from their complex chemical structure.

Chemically speaking, degraders abandon the classical “drug-like” context of Lipinski’s rule-of-five (Ro5) to be considered beyond the Ro5 (bRo5) molecules [9]. More recently, newer guidelines have tried to classify molecules attending to their molecular properties. Starting from Lipinski’s [9] and Weber’s [10] guidelines, Kihlberg et al. [11] suggested several physicochemical guidelines for oral bRo5 drugs including AbbVie’s multiparametric scoring function (AB-MPS) [12]. Moreover, these criteria were later on applied to PROTACs® by Edmondson et al. in 2019 [13], who evaluated several physicochemical descriptors for 40 model PROTACs® and discussed their molecular properties based on their E3 ligases. Additionally, Maple et al. in 2019 [14] developed a study to examine the molecular properties of 422 degraders, establishing a predictive degrader score (Deg_S). Overall, these studies highlight the necessity for property-based resources that enable the identification of degraders with a reasonable potential to become oral drugs [15]. The first attempt in this direction was recently performed by our research group using a pool of 7 rationally selected 2D molecular descriptors (MW, nC, NAR, TPSA, nHAcc, nHDon and PHI) for a dataset of PROTACs® downloaded from PROTAC-DB (http://cadd.zju.edu.cn/protacdb/) [16], an online database exclusively devoted to degraders [17]. Recently PROTAC-DB has been significantly updated and thus the first aim of the study is to provide an overview of this resource. Then, after recalling the concept of chemical space and tailoring to bRo5 molecules [18], we built a PROTAC® chemical space based on the aforementioned descriptors and investigated their distribution in relation to a set of oral available bRo5 drugs. Finally, we identified in this space the position of a small set of PROTACs® for which either bioavailability or permeability has been experimentally determined.

## Experimental

PROTAC®, warhead, linker and E3 ligand csv files were separately downloaded from PROTAC-DB (last download in June 2021) and converted to xlsx files using Microsoft Excel (v. 16.0). Data entries were submitted to OSIRIS DataWarrior Version 5.2.1 (http://www.openmolecules.org/datawarrior/) and alvaDesc 2.0.0 (https://www.alvascience.com/alvadesc/). MW, nC, PHI, nHDon, nHAcc and TPSA(Tot) were calculated with alvaDesc and NAR with Datawarrior. Calculated values were then exported to excel and plotted into 3D graphs with DataWarrior.

## Results and Discussion

### PROTACs® available structures: PROTAC-DB analysis

Common databases, ChEMBL for instance, are expected to enhance drug discovery performance. However, the complexity of PROTACs® (and of most bRo5 compounds) may require bespoke approaches [17]. For instance, the Chemical Probes Portal is starting to include a preliminary dataset of degraders along with their targets to help PROTAC® drug discovery, but it is still very limited (www.chemicalprobes.org). Probes & Drugs (www.probes-drugs.org/home/) have recently updated their content in PROTACs® with data obtained directly from the PROTAC-DB which is an online database fully focused on PROTACs® (http://cadd.zju.edu.cn/protacdb/). This latter introduces for the first time a search engine based on any of the three components of the degrader. Consequently, the search for PROTACs® becomes more user-friendly, in which every PROTAC entry is now considered as an addition of chemical moieties rather than just a fixed chemical structure with a database ID. The PROTAC-DB provides first-hand information about chemical structures, biological activities, and physicochemical properties, retrieved from PubChem, ChEMBL [19], BindingDB [20], and literature. In particular, physicochemical properties are calculated by RDKit (www.rdkit.org) and ALOGPS software [21]. Up to date, 2258 published PROTACs®, 275 warheads, 68 E3 ligands and 1099 linkers are contained in the PROTAC-DB (last download June 2021).

Due to the extensive information provided, regarding public and available information, the PROTAC-DB is the best online source to perform a preliminary analysis of the state of the art in PROTAC® technology. Thus, all the entries including PROTACs®, warheads and E3 ligands were downloaded from the PROTAC-DB. It is necessary to highlight that the number of available entries for each structural group is slightly higher than the number of reported molecular IDs. Therefore, 3939 PROTAC® entries were reported for 2258 PROTACs®, 74 E3 ligand entries for 68 E3 ligand structures and 973 warhead entries for 275 warheads. This fact points out the versatility of some structures within every group, which can indeed target more than one protein (i.e., PROTAC® ID 2199 can be used to target CLK1 or CDK9 proteins). Hence, to have a clear picture of the current trend, PROTAC-DB entries were classified through their targeted protein and their corresponding protein families. Moreover, a count was performed, and the corresponding protein/family clusters were sorted by size.

**Figure 2.**
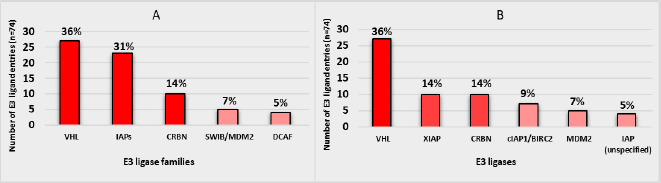
Histogram representation of **A)** E3 ligase protein families and **B)** E3 ligases. Frequencies and percentages are calculated. Values below 5 % for E3 ligase families and E3 ligases are not represented.

**Figure 3.**
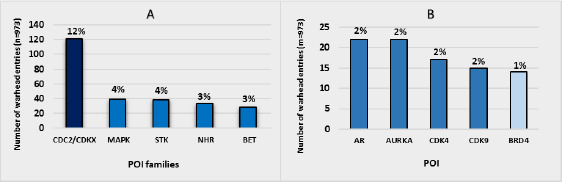
Histogram representation of **A)** POI families and **B)** POI targeted by warheads. Frequencies and percentages are calculated. Values below 3 % for POI families and 1 % for POI are not represented.

The main E3 ligase clusters are represented in frequency histograms (Fig. 2). From the 74 E3 ligand entries, 27 targeted VHL, 23 IAPs and 10 CRBN, revealing that E3 ligands focus mainly on these 3 families of targets (36, 31 and 14 %, respectively) (Fig. 2A). When considering specific E3 ligase proteins (Fig. 2B), VHL remains the most widely used E3 ligase (36 %). Moreover, the IAP family is subdivided into XIAP (14 %), cIAP1/BIRC2 (9 %), cIAP2/BIRC3 (3 %) and unspecified IAPs (5 %), revealing the heterogenicity of this E3 ligase family. Consequently, SNIPERs (Specific and nongenetic IAP-based protein erasers) are rising as degraders using a high variability of E3 ligase proteins [22]. Finally, CRBN (14 %) and MDM2 (7 %) gain the podium of E3 ligase proteins (Fig. 3B). This result reveals the reduced variety of E3 ligases and the limited choice of E3 ligands when designing new PROTACs®. Moreover, despite the higher number of known IAP ligands compared to CRBN ligands, just 1 CRBN E3 ligand, pomalidomide, is used in 1366 out of the 3939 PROTAC® entries (35 %). This fact confirms that CRBN is one of the most used E3 ligases for PROTACs, despite CRBN’s reduced E3 ligand choice range. Consequently, due to the heterogenicity of the IAP family and the great variability of IAP ligands, it is expected that IAP recruiting PROTACs® provide alternative tools in the near future.

Warheads, on the other hand, have a more heterogeneous distribution (Fig. 3) in which 121 warhead entries target the CDC2/CDKX protein kinases subfamily (12 %), 39 MAPK (4 %), 38 Serine/threonine-protein kinases (STK) (4 %), 33 Nuclear hormone receptor (NHR) (3 %) and 28 Bromodomain and extra-terminal (BET) domain family (3 %) (Fig. 3A). Additionally, the Androgen receptor (AR) (2 %), AURKA (2 %), CDK4 (2 %), CDK9 (2 %) and BRD4 (2 %), represent the most targeted proteins, although the distribution is almost equal for all the proteins targeted by warheads. Since the protein of interest will ultimately determine the potential therapeutic use of a degrader, the discovery of new “undruggable” targets justifies this heterogeneous pattern.

**Figure 4.**
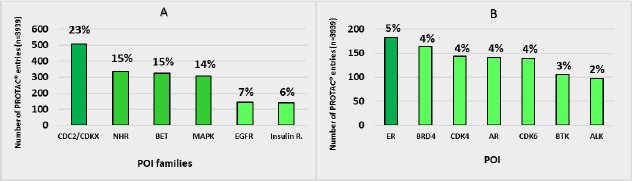
Histogram representation of **A)** POI families and **B)** POI targeted by PROTACs®. Frequencies and percentages are calculated. Values below 6 % for POI families and 2 % for POI are not represented.

Regarding the 3939 PROTAC® entries (Fig. 4), 507 target CDC2/CDKX family proteins (23 %), 336 Nuclear hormone receptors (15 %) 325 target Bromodomain and extra-terminal (BET) domains (15 %), 307 MAP kinases (MAPK) (14 %), 144 Epithelial growth factor receptors (EGFR) (7 %) and 139 the insulin receptor family (6 %), (Figure 4A). In addition, the study reflects that 5 % of published PROTACs® target the Estrogen receptor (ER), 4 % BRD4, 4 % CDK4, 4 % the AR and 4 % CDK6. Moreover, the wide variety of POI targeted by warheads justifies the heterogeneous and scattered POI profile targeted by PROTACs. Notably, PROTAC-DB does not include most proprietary compounds and thus we expect that warheads may cover more POI families.

### One PROTAC® chemical space

The most current definition of chemical space is the ensemble of all possible synthesizable molecules. When defining as bioactive any chemical entity able to specifically interfere with- and alter biological systems, navigating the chemical space to find bioactive compounds sets itself as the primary goal of medicinal chemists. Unfortunately, this is not a trivial task, since the chemical space potentially includes more than 10^180^ molecules, [23]. In order to give a magnitude-order comparison, the biggest structure repository from the American Chemical Society “just” contains 10^8^ structures and only a thousand are bioactive. Moreover, it should be also recalled that bioactivity alone is not sufficient to allow a compound to become a drug and that oral drug delivery is the most preferred administration route. Therefore, most bioactive molecules (often known as chemical probes) do not become oral drug candidates because of the lack of an adequate ADME profile.

**Figure 5.**
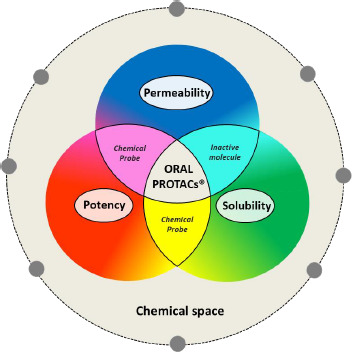
Subregions of the chemical space: schematic representation.

In general terms, PROTACs® occupy a subregion of the whole chemical space that can be called the PROTAC® chemical space. This region includes both PROTACs® with no future as drugs (either inactive molecules or chemical probes) and PROTACs® with potential as oral drug candidates. Therefore, one main aim of pharmaceutical research consists in individuating in the early drug discovery the PROTAC® chemical space subregions enriched in potential drug candidates (Fig. 5).

One reasonable way to do this is to use molecular properties. Molecular properties are quantified by molecular descriptors, which define an n-dimensional chemical space, where n is the number of descriptors considered. Molecular descriptors are in this fashion the orienting coordinates within the chemical space and retrospective analysis of already known compounds individuates regions of interest. In very early drug discovery stages, the chemical matter is not available and therefore in silico descriptors are used at the beginning of any research program. Since all molecular properties fundamentally derive from the atom quality, quantity and connectivity, the first and most fundamental descriptors can be obtained from the molecular formula, such as mass weight, number of atoms, number of specific elements, etc. One level of complexity above this involves the use of atom connectivity-related descriptors. To date, the definition of the classically intended ”drug-like” chemical space (defined by Lipinski’s Rule of five, Ro5) is purely bordered by molecular descriptors calculated from the structure formula, referred as 2D descriptors. Even though the Ro5 still represents the golden region of the chemical space for small molecules, more and more active compounds discovered violate these guidelines. This occurs especially in the case of larger, highly flexible structures, such as PROTACs® [15].

One key aspect of bRo5 compounds is that the larger and more complex a structure is, the more conformation-dependent molecular properties become. Many reports in recent years highlighted the importance of considering the conformational landscape of larger molecules when it comes to defining their molecular properties [24]. Unfortunately, this is not a minor task: with the increase of rotatable bonds number and, consequently, the degrees of freedom, the number of potential conformers increases with factorial function. At present, computational conformational sampling procedures lose accuracy and consistency for bRo5 compounds, making 3D descriptors calculation still challenging.

**Figure 6.**
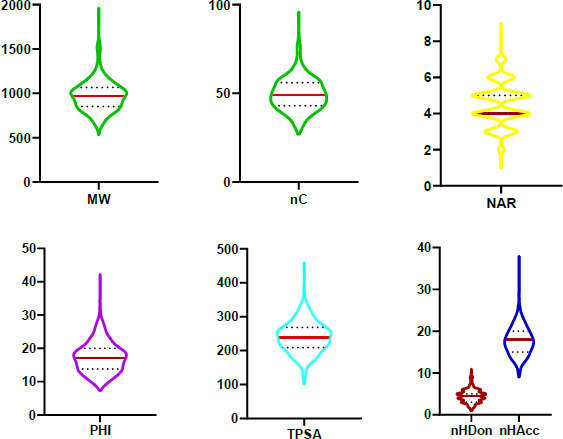
Violin plots representing the molecular property distribution of the 2258 degraders in PROTAC-DB; molecular weight (MW, Da) and number of carbons (nC) (green), number of aromatic rings (NAR)(yellow), Kier’s flexibility index (PHI) (purple), topological polar surface area (TPSA, Å^2^) (light blue) and number of hydrogen bond acceptors and donors (nHDon and nHAcc) (dark red and blue, respectively). The 50th percentiles or medians are represented as solid horizontal bars and the 25th and 75^th^ percentiles as dashed lines.

Therefore, due to the inherent conformational limitations of PROTACs®, we selected a pool of 2D descriptors that provide an unequivocal starting point in the definition of PROTAC® chemical space [17]: MW as a descriptor of molecular size; a set of count descriptors related to both polar (nHAcc (also HBA), nHDon (also HBD)) and nonpolar (the number of carbon atoms, nC and the number of aromatic rings, NAR) molecular moieties; a flexibility descriptor (PHI) [25] and TPSA as a polarity index. In practice, we set up a pool of seven descriptors, three of nonpolar nature, three of polar nature and one flexibility descriptor. Figure 6 shows violin plots representing the seven molecular property distribution of the 2258 degraders in PROTAC-DB (average values are: MW= 972.9 Da, nC= 49.5, NAR= 4.5, PHI= 17.3, nHDon= 4.5, nHAcc= 17.7 and TPSA= 240 Å^2^) (Table S1).

**Figure 7.**
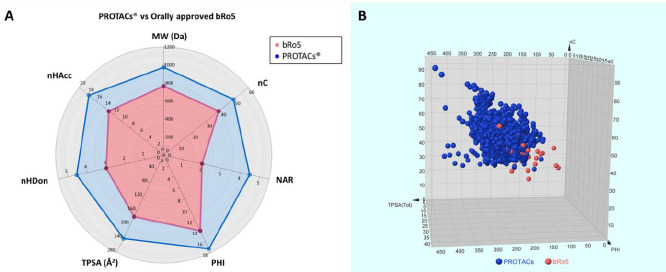
**A)** Radar plot comparing the seven 2D descriptors for degraders and orally approved bRo5 drugs **B)** Chemical space occupied by PROTACs® and bRo5 approved drugs.

The average of the 7 calculated descriptors for degraders were compared to those previously calculated for 50 orally approved bRo5 drugs [17] (MW= 765 Da, nC= 39, NAR= 2, PHI= 14, TPSA= 177 Å^2^, nHDon= 3 and nHAcc= 13), introduced by Kihlberg et al. [18]. Results, presented in a radar plot, confirm that PROTACs® cover a wider property range, being the degrader’s NAR more than twice the value for orally approved bRo5 drugs (Fig. 7A). Nevertheless, it must be mentioned that some of the represented PROTACs® were designed as scientific tools, unintended to be pharmaceutical candidates with optimal DMPK properties. Moreover, to better visualize the property distribution we defined a chemical space by including three representative descriptors: nC (nonpolar), TPSA (polar) and PHI (flexibility) (Fig. 7B).

The picture emerging is that the PROTAC® chemical space is adjacent and only partially stackable to the region occupied by oral bRo5 drugs: the majority of PROTACs® have a higher number of carbon atoms than the analysed bRo5 compounds. This is a bona fide indicator of larger structures. Topological polar surface area is also higher. This aspect is of greater interest since a greater polar surface area is often associated with lower permeability. Nevertheless, here we are considering fragment-calculated (topological) 2D surface area which does not include 3D information. Thus, this aspect must be carefully considered. Indeed, PROTACs® present in PROTAC-DB show also a higher Kier’s flexibility index (PHI), suggesting that the average increase in polar fragments, might be compensated with higher flexibility and polar moieties shielding. This fact has already been extensively reported in the bRo5 chemical space [26,27]. Finally, it should be recalled that PROTAC-DB does not include most of the proprietary compounds which might occupy further regions in the chemical space.

### Subregions in the PROTACs® chemical space populated with potential oral drugs

**Figure 8.**
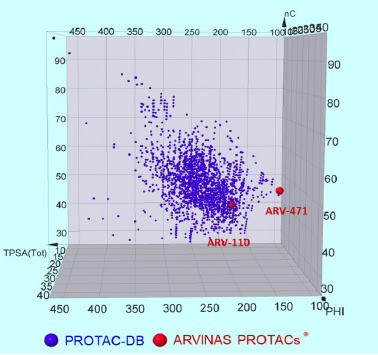
Chemical space occupied by ARV-110 and ARV-471.

A PROTAC® chemical space represents an extraordinary tool to identify groups of degraders or clusters sharing similar chemical or in vitro ADME properties. In particular, the potential of degraders to become oral drugs could be defined by bioavailability and permeability, among others. Bioavailability (F%) is defined as the fraction of drug that reaches systemic circulation and thus needs an in vivo experimental determination. Consequently, the recently disclosed orally bioavailable degraders ARV-110 and ARV-471 (Fig. 8), were introduced into the chemical space defined above. Both structures are located in the boundaries of the chemical space. Regarding their molecular size, their structure contains 41 and 45 carbons respectively, being placed on the first quartile with respect to the PROTAC-DB average (nC= 49.5) (Fig. 6). Moreover, their significantly low PHI index (12 and 10 respectively) compared to the PROTAC-DB average (PHI= 17.3) places both degraders in the first quartile (Fig. 6). Both structures incorporate a semi-rigid linker 1-[(piperidin-4-yl)methyl]piperazine (chemical structures in Fig. S1), which compared to traditional pegylated or alkyl linkers, are less flexible. Considering the importance of flexibility to the formation of intramolecular hydrogen bonds (IMHBs), these two degraders occupy a subregion where, at least theoretically, dynamic IMHBs are not favored [28] (a dynamic IMHB is formed in nonpolar but not in polar media). Consequently, this structural property profile reduces Arvinas degrader’s capacity to change conformation in an environment-dependent manner, that is, to behave as molecular chameleons. In addition, ARV-110 and ARV-471 have a TPSA value of 182.86 Å^2^ and 96.43 Å^2^, respectively, which places them in the first quartile (TPSA average= 240 Å^2^). Overall, after analysing their properties it could be stated that both degraders occupy a superimposable region to that of oral bRo5 drugs: (nC: 40-50, PHI: 10-15 and TPSA: 50-200 Å^2^) (Fig. 7). Thus, this drug design strategy seems to have prioritized candidates staying as close to the Ro5 chemical space as possible.

**Figure 9.**
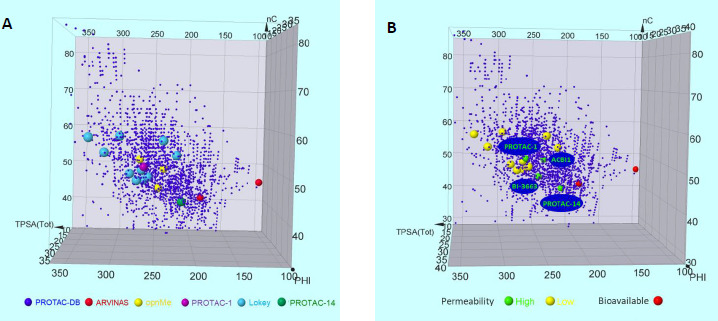
Chemical space of a **A)** Subset of permeable degraders **B)** Graphical distribution of highly permeable and bioavailable degraders.

Permeability is one of the major determinants of bioavailability. It is physiologically defined as the ability of a compound to pass across biological membranes and is determined *in vitro* with different methods, PAMPA and Caco-2 being the most common ones (a discussion about the differences between the two methods and their reliability when applied to bRo5 compounds is beyond the aim of the paper [28,29]). However, the limitations of obtaining reliable permeability data for large molecules [27] suggest that degraders with an apparent low passive permeability could surprisingly display decent bioavailability values. For this reason, a set of degraders showing consistent permeability values were identified: PROTAC-1 described by Kihlberg et al. [30], a small set provided for free by opnMe (www.opnme.com) [25], PROTAC-14 by Skidmore et al. [31] and 11 degraders reported by Lokey et al. [32] (Figure S1 and Table S2). These degraders were then located into the chemical space (Fig. 9A) and classified into permeable (*P*_app_ > 1 x 10^-6^ cm/s) or low permeable (*P*_app_ < 1 x 10^-6^ cm/s) (Fig. 9B) (Table S2). Notably, permeable degraders (PROTAC-1, PROTAC-14, ACBI1 and BI-3663) occupy a central region of the chemical space (Figure 9b). Among them, BI-3663 and PROTAC-14, are the closest to Arvinas’s compounds. In particular, PROTAC-14 is the degrader showing the best “drug-like” properties (Table S3). It has the structure containing less nC and the lowest PHI and TPSA values. Moreover, BI-3663 is slightly more polar than ACBI1 but has 5 carbons less and thus is closer to ARV-110 and ARV-471. Finally, PROTAC-1 has a higher nC (50), TPSA (265 Å^2^) and PHI (19) which places it above the other degraders. PROTAC-1 is permeable although large dimensions and high TPSA are associated with low permeability. However, PROTAC-1 is highly flexible and thus capacity to form dynamic intramolecular interactions that confer it the capacity to behave as a molecular chameleon [30]. Chameleonicity therefore could explain why PROTAC-1 is permeable despite its position in a less “drug-like” region of the PROTAC® chemical space.

Overall, our analysis suggests that permeable PROTACs® could be located in three regions: 1) the subregion having the “best drug” like properties (smaller size, lower polarity and flexibility) (PROTAC-14), 2) an intermediate region with acceptable molecular properties (BI-3663 and ACBI1) and 3) a region occupied by molecular chameleons (PROTAC-1). However, since chameleonicity cannot be only explained on the basis of 2D descriptors (the determinants of chameleonicity are beyond the scope of this paper) it is possible that molecular chameleons with optimal permeability could occupy further regions of this 2D property-based chemical space.

## Conclusions

PROTACs® are an additional modality to the drug discovery toolbox and ad hoc strategies to fully exploit their potential as oral drugs are still under study. In this paper, we first explored the PROTAC-DB which represents a useful tool for degrader design. In particular, we identified the most popular targets in degrader technology and calculated statistically their distribution. E3 ligands have been revealed to target mainly three E3 ligase families (VHL, IAPs and CRBN) that represent up to 81 % of the total. Warheads, on the contrary, show a heterogeneous distribution in which only 2 % target the same POI. This high warhead variability confers PROTACs® the capacity to degrade multiple targets, being the ER the most popular target (5 %).

The updated version of PROTAC-DB allowed us to update and expand the PROTAC® chemical space previously described by some of us [17] and based on a set of seven calculated 2D descriptors (MW, nC, NAR, PHI, nHDon, nHAcc and TPSA). Then we compared the PROTAC® with an orally approved bRo5 drugs chemical space and provided for the first-time initial insights on the location of bioavailable (ARV-110 and ARV-471) and a small dataset of permeable PROTACs®. Results show that bioavailable degraders are close to the oral bRo5 region whereas permeable PROTACs® seem to occupy an extended and central region of the chemical space. In addition, the fact that chameleonicity can improve permeability could locate molecular chameleons towards the less “drug-like” region of the chemical space.

Overall, we proved that the chemical space using nC, TPSA and PHI helps to identify regions of PROTACs® with future as oral drugs if permeability/bioavailability data are available. Since limitations associated to the measurement of bRo5 permeability are known, the determination of experimental physicochemical descriptors able to predict permeability could accelerate the identification of new oral PROTACs®. Studies along these lines are in due course in our laboratories.
