# Supplementary material for: Are we ready to design oral PROTACs®?
Source: ADMET DMPK. 2021 Aug 31;9(4):243–54. doi: 10.5599/admet.1037 (PMC8920102; doi:10.5599/admet.1037)

Supplementary information

Are we ready to design oral PROTACs®?

Diego Garcia Jimenez, Matteo Rossi Sebastiano, Giulia Caron, Giuseppe Ermondi*

University of Torino, Molecular Biotechnology and Health Sciences Dept., CASSMedChem, via Quarello 15, 10135 Torino, Italy.

Table S1: Statistical results of the 2D descriptors in PROTAC-DB.

	MW	nC	NAR	PHI	nHDon	nHAcc	TPSA(Tot)
**Number of values**	2258	2258	2258	2258	2258	2258	2258
**Minimum**	533.6	27.00	1.000	7.357	1.000	9.000	101.2
**25% Percentile**	851.8	43.00	4.000	13.83	3.000	15.00	209.1
**Median**	970.1	49.00	4.000	17.05	4.500	18.00	239.0
**75% Percentile**	1067	56.00	5.000	20.03	5.000	20.00	268.5
**Maximum**	1963	96.00	9.000	42.27	11.00	38.00	460.1
**Range**	1430	69.00	8.000	34.91	10.00	29.00	358.8
**Mean**	972.9	49.52	4.484	17.32	4.539	17.70	240.0
**Std. Deviation**	174.1	9.270	1.219	4.622	1.590	3.372	45.97
**Std. Error of Mean**	3.664	0.1951	0.02565	0.09727	0.03345	0.07096	0.9674

Table S2: Permeable PROTACs® introduced in the chemical space. Permeability values and their assesment methods. Caco-2: (AB: apical-basal, BA: basal-apical)

Name	Permeability (10^{-6} cm/s)	Permeability experiment	Permeability classification	Source
ACBI1	2	Caco-2	High	opnMe
BI-0319	0.2	Caco-2	Low	opnMe
BI-3663	15.85	Caco-2	High	opnMe
BI-4206	0.4	Caco-2	Low	opnMe
PROTAC-1	1.41	PAMPA	High	Kihlberg
PROTAC-14	1.7 (AB), 14.1 (BA)	Caco-2	High	Skidmore
MZ4	0.60	PAMPA	Low	Lokey
MZ1	0.03	PAMPA	Low	Lokey
MZ3	0.01	PAMPA	Low	Lokey
MZP-61	0.30	PAMPA	Low	Lokey
MZP-55	0.16	PAMPA	Low	Lokey
CM09	0.01	PAMPA	Low	Lokey
CM11	0.008	PAMPA	Low	Lokey
CMP98	0.00	PAMPA	Low	Lokey
AT3	0.01	PAMPA	Low	Lokey
AT6	0.00	PAMPA	Low	Lokey
AT2	0.00	PAMPA	Low	Lokey

Table S3: Calculated 2D descriptors for highly permeable and bioavailable PROTACs® introduced in the chemical space.

Name	MW	nC	NAR	PHI	nHDon	nHAcc	TPSA(Tot)
ARV-110	812.38	41.00	3.00	11.97	2.00	16.00	182.86
ARV-471	723.99	45.00	4.00	9.88	2.00	9.00	96.43
ACBI1	936.23	49.00	5.00	15.04	6.00	17.00	236.84
BI-3663	917.93	44.00	4.00	15.56	4.00	22.00	244.47
PROTAC-1	1034.24	50.00	5.00	19.04	4.00	22.00	264.87
PROTAC-14	837.9	40.00	3.00	13.73	1.00	18.00	210.82

Figure S1: Chemical structures for highly permeable and bioavailable PROTACs® introduced in the chemical space.

ACBI1

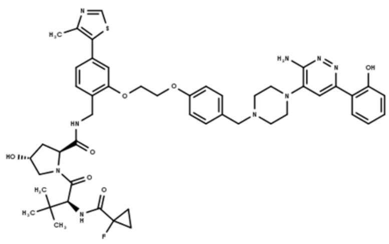

PROTAC-1

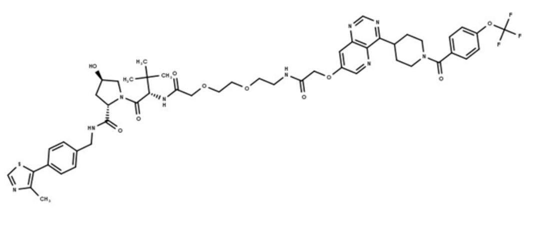

BI-3663

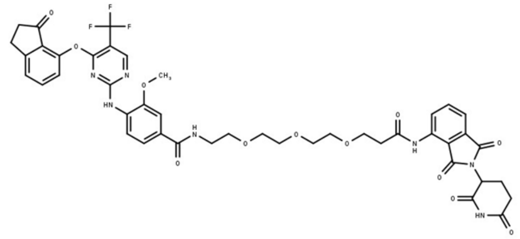

PROTAC-14

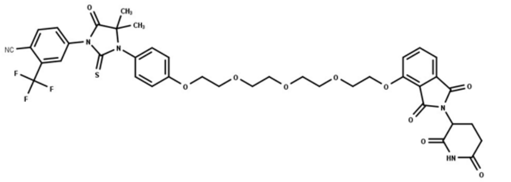

Supplement: Supplementary file 1 [file admet-9-1037-S1.pdf]
